# Trainees4trainees: an innovative peer support project for junior doctors across specialties

**DOI:** 10.1192/bjo.2021.363

**Published:** 2021-06-18

**Authors:** Sophie Behrman, Aisling Higham, Haido Vlachos, Gerti Stegen

**Affiliations:** 1 Oxford Health NHS Foundation Trust; 2 Oxford University Hospitals, NHS Foundation Trust; 3 Health Education England – Thames Valley

## Abstract

**Aims:**

The BMA's survey results (Caring for the Mental Health of the Medical Workforce, 2019) and HEE's NHS Staff and Learners’ Mental Wellbeing Commission report (2019) highlighted declining staff wellbeing. The COVID-19 pandemic has sharpened focus on this and the effects of moral injury on healthcare professionals. Shielding, social distancing and redeployment led to many medical trainees being increasingly isolated at a time of heightened anxiety and adversity. Psychiatry trainees tend to have good access to reflective groups, but this is not customary in other training programmes.

**Method:**

Intervention

“Trainees4trainees” was set up by trainees across specialties as a HEE-TV well-being project, led by the Deanery Trainee Improvement Fellow. Peer support groups are run on Zoom, facilitated by 2 trainees with special training in peer support. Psychiatry trainees have been involved in designing and facilitating groups and training facilitators from other specialties; facilitators have regular supervision from a consultant psychiatrist in medical psychotherapy. Trainees are supported to discuss challenging experiences and think about their emotional responses in a supportive and validating group.

**Result:**

Feedback

We are in the process of formal data collection to assess the impact of the intervention. Informal feedback suggests the groups are a powerful support to individuals who otherwise have no avenue to think about the psychological impact of their experiences. The groups have supported trainees to feel less isolated and bolstered their resilience.

**Conclusion:**

Future plans

We have faced challenges in the practicalities of establishing and maintaining groups. We are working with Training Programme Directors to move towards running the groups in protected time within working hours and advocate that reflective groups, such as our peer support groups, are a key part of future medical and surgical Training Programmes.

